# The Determination of Type 2 Diabetes Mellitus's Impact on the Density of Retinal Blood Vessels and the Choriocapillaris: Optical Coherence Tomography Angiography Study

**DOI:** 10.1155/2021/7043251

**Published:** 2021-11-22

**Authors:** Marko Zlatanović, Jasmina Đorđević Jocić, Vesna Jakšić, Nevena Zlatanović, Mlađan Golubović, Maja Živković

**Affiliations:** ^1^Clinical Center Niš, Ophthalmology Clinic, Niš, Serbia; ^2^University of Niš, Faculty of Medicine, Department of Ophthalmology, Niš, Serbia; ^3^University of Belgrade, Medical Faculty, Department of Ophthalmology, Belgrade, Serbia; ^4^Community Health Center Niš in Niš, Niš, Serbia; ^5^University of Niš, Medical Faculty, Clinic for Anesthesiology and Intensive Care at the Clinical Center Niš in Niš, Niš, Serbia

## Abstract

Optical coherence tomography angiography (OCTA) was used to analyze the alterations in the density of retinal blood vessels and the choriocapillaris (VD) in patients suffering from type 2 diabetes mellitus (T2DM). One hundred sixty-six eyes of 83 patients (43 of whom were men and 40 women, with a mean age of 58.59 ± 14.04) with T2DM and without diabetic retinopathy were examined for the purpose of conducting the observational prospective study. The control group (CG) consisted of 66 eyes in 33 healthy subjects (15 male and 18 female, with a mean age of 55.12 ± 12.70). The measurement regions of vessel density (VD) included the deep capillary plexus (DCP), the superficial capillary plexus (SCP), and the choriocapillaris. The results indicate considerable differences in the VD of the DCP and SCP when comparing the control group with the study groups (*p* < 0.001). In comparison with the control group (*p* < 0.001), there was a statistically significant reduction in the VD of the choriocapillaris in the study group. Furthermore, patients with T2DM showed a significantly decreased VD concerning the control in different macular regions. Thickness in several macular regions in the study group significantly decreased compared to the ones in the control group. OCTA was used to gather relevant information about the vascular changes which occurred in T2DM patients, assessed through the quantitative analysis of the blood flow in the retina and choriocapillaris.

## 1. Introduction

Type 2 diabetes mellitus, a metabolic disease of a complex and multifactorial nature, is characterized by the incapability of cells to level up glucose levels in the blood, as well as insulin resistance, leading to hyperglycemia, the main cause of diabetic complications [1–3]. Among many diabetic complications, one of particular interest is diabetic retinopathy (DR) [4–6]. Depending on several DR clinical features, which include hard exudates, cotton wool spots, macular edem, neovascularization hemorrhages, and microaneurysms, the classification of DR is twofold: (a) nonproliferative diabetic retinopathy (NPDR) and (b) proliferative diabetic retinopathy (PDR). NPDR, further divided into stages that can be mild, moderate, or severe, is a phase during which the retina does not show any new blood vessel growth. Unfortunately, DR is identified to be among the main causes of the loss of eyesight, and if not treated, DR will lead to visual impairment that cannot be regained; therefore, identifying microvascular changes before the clinical signs of DR become apparent at its early stage is preferable to action for preventing vision impairment [7, 8].

With the aim of the quick creation of angiographic images, optical coherence tomography angiography (OCTA) uses information obtained from motion contrast imaging to high-resolution volumetric blood flow and is considered a noninvasive imaging technique. OCTA compares the decorrelation signals, defined as the differences in the amplitude or the intensity of the OCT signal that is backscattered, occurring between sequential OCT b-scans, and these must be performed at an identical cross section. In this manner, OCTA forms a blood flow map, strictly representing the movement of erythrocytes in the blood vessels of the retina [9–15].

The main aim of this study is to determine what kind of impact type 2 diabetes mellitus has on the density of the blood vessels of the retina and choriocapillaris with the application of optical coherence tomography angiography.

## 2. Methods and Materials

The present study involves the examination of two hundred and thirty-two eyes of 116 patients without DR, who reported to the Ophthalmologic Clinic, Clinical Center Niš, between March and July 2018. The inclusion criterion was age, ranging from 40 to 70, with both genders equally included. The exclusion criteria were age-related macular degeneration, vascular diseases of the retina, glaucoma, myopia exceeding 6 diopters, history of panretinal laser photocoagulation after eye surgery, as well as other types of eye disorders, intravitreal administration of any drug, and significant lens opacity, for the purpose of avoiding reduced-quality OCTA images. Sixty-six eyes belonging to 33 healthy subjects that were matched by age (15 men and 18 women, with a mean age of 55.12 ± 12.70) whose ophthalmological examination yielded normal results and no eye diseases were used as the control group. The group of patients included 83 patients (166 eyes) suffering from type 2 diabetes mellitus without DR. Each patient went through a full eye exam, which included the following examinations: slit-lamp biomicroscopy, fundus examination, fundus autofluorescence (FAF), applanation tonometry, best-corrected visual acuity (BCVA) based on the Early Treatment of Diabetic Retinopathy Study (ETDRS), and OCTA (RTVue XR Avanti, AngioVue, Optovue, Inc., Freemont, CA). The participants in the study gave their informed written consent.

All the investigations respected the principles of the Declaration of Helsinki, and the Ethical Committee of the Faculty of Medicine, University of Niš, gave their approval for the mentioned.

### 2.1. Optical Coherence Tomography Angiography

The Optovue AngioVue System (ReVue XR software version 2017.1.0.151, issued by Optovue Inc., Fremont, CA, USA) was used to obtain OCTA images after conducting the standardized protocol whose basis is the SSADA (split-spectrum amplitude decorrelation algorithm) as detailed in the literature [12]. The classification of diabetic retinopathy according to the ETDRS was used as guidance for macular capillary network visualization, with scans performed centered on the fovea area (3 mm × 3 mm) over the macular region divided into fovea, parafovea, and the full image. The automatic vascular network segmentation of the deep capillary plexus (DCP), superficial capillary plexus (SCP), and the choriocapillaris was performed with the application of the AngioAnalyticTM software. Vessel density (VD) was calculated by the same software and represented as the percentage of the microvasculature-occupied area and thickness in the total area of the scan and all of the sections. In this study, the definition of the choriocapillaris VD is the flow area divided by the selected area (radius was set to 1.5 mm). Software that makes use of the 3D Projection Artifact Removal (PAR) algorithm was used for the purpose of improving OCTA image quality. We excluded from the analysis images with residual motion artifacts, low centration and focus, incorrect segmentation, and the index of the signal strength below 40.

### 2.2. Statistical Analysis

The statistical analysis was done with SPSS 15 (SPSS Inc., Chicago, IL) for Windows. The Kruskall–Wallis test was used to analyze nonparametric data. The unpaired t-test was applied for the purpose of comparing the OCTA parameters between the control and test groups. It is considered that the *p* value of <0.05 was of statistical significance.

## 3. Results

This study included 166 eyes of 83 patients (43 men (51.8%) and 40 women (48.2%)) with a confirmed diagnosis of diabetes. The average age of the subjects in the diabetes group was 58.59 ± 14.04 (the mean age of the women was 59.10 ± 15.20, and that of the men was 58.10 ± 12.92). Thus, men and women were practically the same age (*p*=0.652). The control group (CG) consisted of 66 eyes in 33 subjects, of whom 15 (45.5%) were men and 18 (54.5%) were women. The average age of the subjects in this group was 55.12 ± 12.70 (the mean age of women was 55.28 ± 12.71, and that of men was 54.93 ± 12.91). The men and women were of the same age (*p*=0.914). The frequency of male respondents among patients with diabetes was 51.2%, and in the CG, it was 45.5%. 48.8% of the female respondents suffered from diabetes, and 54.5% had it in the CG. According to the proportion of male and female respondents, the groups (the group of patients and the CG) did not differ (*p*=0.466).

According to the obtained results, retina superfical VD exhibits a much lower value (*p* < 0.001) in the group of patients (41.9 ± 6.8) than in the control group (46.9 ± 3.9). Retina deep VD is affected by whether or not the percipient has diabetes, since in the patient group, the mean value (48.1 ± 5.0) is statistically significantly lower (*p* < 0.001). Whether the patient has diabetes or not, does not significantly affect the change in retinal thickness for both the superfifical and the deep. The values obtained from the statistical analyses of the other obtained OCTA parameters are presented in Table 1.

The observation of the mean values of the VD of individual sectors of retina superficial showed that only the value of the fovea was the same both in the group with patients and in the control group. All other sectors—parafovea (45.0 ± 5.8), superior hemi (44.8 ± 6.1), inferior hemi (45.2 ± 5.8), tempo (43.6 ± 6.3), superior (46.0 ± 6.4), nasal (44.7 ± 6.0), and inferior (46.2 ± 6.2)—show a significantly reduced (obtained *p* value was <0.001) values compared to the control group, where the average values were the following: parafovea (50.1 ± 4.2), superior hemi (49.5 ± 5.4), inferior hemi (49.9 ± 4.7), tempo (48.2 ± 4.1), superior (50.7 ± 5.4), nasal (49.6 ± 4.8), and inferior (51.5 ± 4.8).

**Figure 1:**
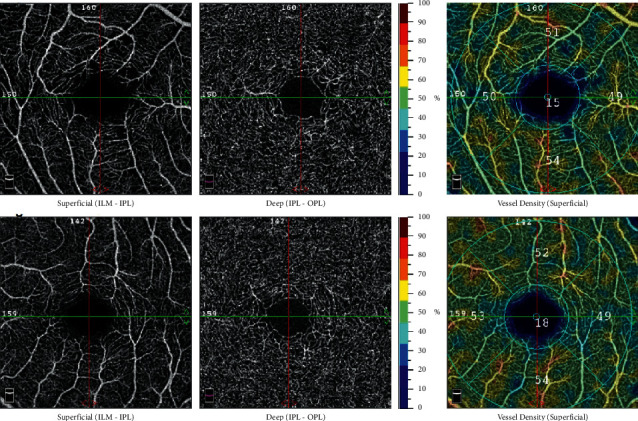
Optical coherence tomography angiography (OCTA) of the retina from a patient suffering from type 2 diabetes (a) and a subject who is healthy (b).

There is no considerable difference of the average values of the retina deep fovea sector when we compare the group with patients and the control group, as is the case with the same retina superficial sector. All the other sectors—parafovea (50.1 ± 5.5), superior hemi (50.1 ± 6.6), inferior hemi (49.8 ± 5.6), tempo (49.9 ± 5.6), superior (50.6 ± 6.4), nasal (50.4 ± 6.5), and inferior (49.3 ± 6.5)—have a much more reduced (obtained *p* value was <0.001) value than those of the control group does, which exhibits the following values: parafovea (55.8 ± 2.9), superior hemi1 (55.9 ± 3.0), inferior hemi (55.8 ± 3.1), tempo (55.5 ± 3.0), superior (56.1 ± 3.3), nasal (56.0 ± 2.7), and inferior (55.5 ± 3.7). The images obtained from OCTA regarding the VD of a patient with T2DM and a healthy individual are shown in Figure 1.

**Figure 2:**
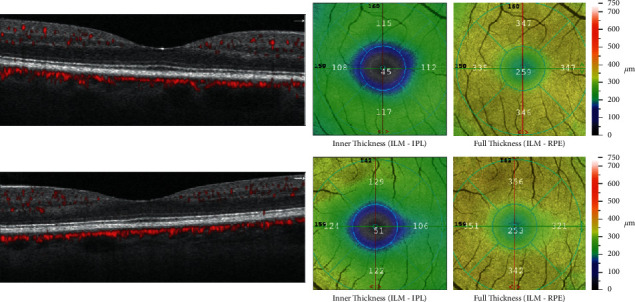
The thickness of the retina obtained by OCTA from a patient suffering from type 2 diabetes (abovementioned) and a subject who is healthy (below).

There were no statistically significant differences in all the average values of the retinal thickness sectors between the CG and the patient group. The images obtained from OCTA regarding the thickness from a patient with T2DM and healthy individuals are shown in Figure 2.

**Figure 3:**
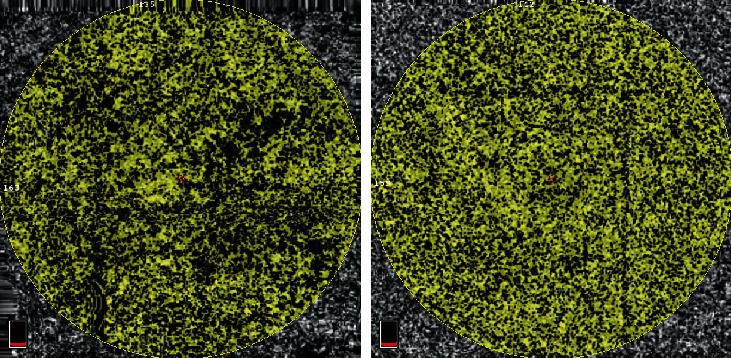
Choriocapillaris obtained by OCTA of the retina from a patient suffering from type 2 diabetes (a) and a subject who is healthy (b).

According to the obtained results regarding choriocapillaris, the flow area was much lower (*p* < 0.001) in the patient group (4.622 ± 0.389) than that in the control group (4.791 ± 0.252). The same observation was valid for choriocapillaris VD, where the stated parameter was much lower (*p* < 0.001) in the patient group (0.642 ± 0.097) than that in the control group (0.678 ± 0.035). The images obtained from OCTA regarding choriocapillaris from a patient with T2DM and healthy individuals are shown in Figure 3.

## 4. Discussion

Although DR is treated as a complex condition, recent studies show that DR could be related to microvascular abnormality development [16, 17]. For this reason, microvascular abnormality development in the vascular layers of the retina was evaluated in this study with the application of OCTA, and it was indicated with superﬁcial and deep VD. Previous studies suggested that a combination of reduced VD, accompanied by increased FAZ area, could be considered as parameters used to detect DR at an early stage [18, 19]. Lupidi et al. published a comparative analysis that compared groups of diabetics and healthy people, demonstrating differences that are of a statistical significance considering the major axis, surface, and perimeter of the FAZ, owing to the FAZ enlargement found in diabetic maculopathy, both of superficial and deep capillary plexuses, suggesting that progressive nonperfusion was not limited to a particular layer in diabetic patients [20].

The findings reported by Dimitrova et al. show that the density of superficial capillaries and deep capillaries has decreased in the parafoveal area of the retina and the choriocapillaris in T2DM patients that do not have DR [19]. In addition, Simonett et al. reported that there is a notable decrease in the density of the parafoveal vessel within the deep capillary plexus, detected in patients suffering from T1DM and those that have mild DR [21]. Our study supports these findings since we have discovered that there is a significant difference in the VD parameters of the retina superficial and the retina deep, both exhibiting lower values in the patient group. This process could be associated with the larger extent of microaneurysms in the deep vascular plexus than those in the superficial plexus [22–25]. Previous studies related to retinal thickness, based on OCT, presented controversial results, such as the one where no significant difference was found in the thickness concerning both the group of patients and healthy controls, and another where there was a notable reduction in thickness present in patients suffering from diabetes without DR or those with mild NPDR when compared to the healthy population [26, 27]. Our study does not reveal any significant difference difference in the OCTA parameters related to retina thickness between the groups.

According to Lupidi et al. subjects suffering from diabetes were observed to have a significant negative linear correlation between CC VPD and DCP VPD. However, the correlation between CC VPD and SCP VPD was insignificant. The authors reported further that there was a positive linear correlation between SCP, or CC VPD and DCP VPD, in healthy subjects. According to the obtained results, Lupidi et al. suggested that there could be a functional interconnectedness between the choroidal vascular networks and those of the retina based on the hypothesis that, in terms of VPD, the negative linear correlation between DCP and CC could express a potential mechanism for compensation [28]. The DCP-level blood flow may slightly increase for the purpose of supplying the outer layers of the retina in the event of CC insufficiency, while the CC perfusion may increase with the aim of compensating for the demand for oxygen and the metabolic demand in case retinal capillary impairment occurs, which often includes the DCP in the case of DR [18, 29].

Based on the current information at our disposal, we believe that no other study has observed changes in the different parts of both the superficial and the deep retina (parafovea, fovea, inferior hemi, superior hemi, tempo, inferior, superior, and nasal). According to the obtained results for both superficial and deep retina, only the value of the fovea is the same both in the group of patients and in the control group. The value for retina thickness did not differ between the patient and healthy population groups. The complex nature of diabetes and both vascular and neurodegenerative changes prevents us from giving a possible explanation for this.

Different studies related to choriocapillaris reported controversial findings. Impairments of the focal and diffuse CC flow in the eyes of patients suffering from diabetes were reported by Choi et al. while Nesper et al. [30, 31] reported an increased CC nonperfusion area in the eyes of the patients suffering from diabetes without retinopathy, compared to the ones of the control group. Other studies [19, 32] have reported no notable differences in the density of the CC vessel when comparing eyes of diabetics without retinopathy and those of healthy controls. According to the findings presented in this research, both the choriocapillaris flow area and the choriocapillaris VD were much lower in the group of patients than in the control group, and these results are supported by previous histopathological studies [33].

The presented study has several limitations related to a single-center analysis (all subjects were of the same ethnicity) and single time-point (data were taken once; therefore, the evaluation of changes over time is not possible).

In summary, the results presented in this study indicate that OCT angiography parameters associated with the density of the superficial and deep vessels of the retina have lower values in patients suffering from diabetes without DR than in healthy subjects. One of the reasons for these differences could be the autoregulation alternation of the retinal blood vessels in patients suffering from diabetes without DR, further associated with a patient's systemic characteristics. According to the obtained results, OCT angiography could be considered as a potential biomarker used for the risk of DR development in patients suffering from diabetes without DR evaluation.

## Figures and Tables

**Table 1:** Differences vessel density between the control and patient groups obtained with OCTA.

	Density				Thickness	
	Retina superficial		Retina deep			
	Control group	Patient group	Control group	Patient group	Control group	Patient group
Fovea	17.5 ± 7.7	17.0 ± 7.5	31.3 ± 7.1	32.2 ± 7.9	324.0 ± 29.4	327.6 ± 10.8
Parafovea	45.0 ± 5.8	50.1 ± 4.2	50.1 ± 5.5	55.8 ± 2.9	321.3 ± 23.2	327.6 ± 10.8
Superior hemi	44.8 ± 6.1	49.5 ± 5.4	50.1 ± 6.6	55.9 ± 3.0	322.7 ± 23.9	328.3 ± 12.5
Inferior hemi	45.2 ± 5.8	49.9 ± 4.7	49.8 ± 5.6	55.8 ± 3.1	319.4 ± 23.1	326.3 ± 12.2
Tempo	43.6 ± 6.3	48.2 ± 4.1	49.9 ± 5.6	55.5 ± 3.0	315.5 ± 22.4	318.4 ± 10.3
Superior	46.0 ± 6.4	50.7 ± 5.4	50.6 ± 6.4	56.1 ± 3.3	324.9 ± 24.5	332.1 ± 11.7
Nasal	44.7 ± 6.0	49.6 ± 4.8	50.4 ± 6.5	56.0 ± 2.7	325.0 ± 26.2	329.5 ± 14.9
Inferior	46.2 ± 6.2	51.5 ± 4.8	49.3 ± 6.5	55.5 ± 3.7	319.8 ± 24.9	327.3 ± 14.6

## Data Availability

The authors confirm that the data supporting the findings of this study are available within the article.
